# The Cultivation of Legacies: Help the Lawyers to Help the Hospitals

**Published:** 1918-05-25

**Authors:** 


### THE CULTIVATION OF LEGACIES.

### Help the Lawyers to Help the Hospitals.

THE war has revolutionised many things and amongst them stereotyped methods in voluntary hospital business. On the whole the financial side of the voluntary hospitals in the years during which the war has prevailed has proved encouraging. Indeed in those cases where the administration is up to date, highly intelligent, and watchful, the yield in income shows increases under nearly every head of revenue. One of the most remarkable instances of this will be found in the last audited statements of the North Staffordshire Infirmary, Hartshill, *i.e.* for the year ending October 25, 1917. One reason for this general tendency no doubt is that the advent of war has entailed the necessity of examining into each source of income, with encouraging results. Nevertheless, taking voluntary hospitals as a whole we should say, as a matter of long experience, that only a relatively small minority of the whole number obtain through legacies the yield which should be their share.

The department devoted to finance should be under the guidance of the most capable and influential supporters of each hospital. Too many hospitals leave what is popularly called a detail of this description to their secretary, and he, poor man, in the present day, with all the other contingent duties, has really not the time or the influence or the opportunity to reap in fields from which large contributions and legacies can, under proper conditions, be gathered for a particular hospital. The securing of legacies necessitates the keeping of the name of a particular hospital before those who have to do with members of the public in possession of adequate means, a portion of which may be available for distribution in due course in the form of legacies. The keeping of a hospital constantly before the public and under public notice makes its name familiar to a host of people who may never enter its doors or during their life take any part in its support or management. If that hospital deals mainly with the treatment of a particular disease, a steady yield of legacies is sure to result, and it would not be difficult to name some where the annual sum received from this source is far and away larger than the particular institution can properly lay claim to by reason of its size or needs or work.

We mention this because too many hospital committees and others seem to think that advertising is a waste of money, and that publicity costs too much to warrant them devoting time to its mastery. We venture to call the attention of the hospital world to the cultivation of legacies because a gentleman of great influence in a provincial centre, where he was the leading representative of the law, sought an introduction to express his indebtedness to the Editor for what he described as the invaluable and remarkable book, "Burdett's Hospitals and Charities." He said that book had become the bible of many lawyers whose clients were people of position and large means, and that they really could not do without it. It had become indispensable in fact. He wished to have a little further help by having within ready reach of the testators a Solicitors' List of Voluntary Charities, stating in the tersest way their immediate and pressing needs from month to month.

It appeared that many legacies had passed through his hands to voluntary hospitals, and he had made a study of the subject. In the result we promised to try to supply his needs through THE HOSPITAL. The proposal is to give up each week a page or more of THE HOSPITAL to those hospitals which may wish to try to cultivate legacies on the plan this solicitor suggests. The page will be devoted exclusively to those hospitals which decide deliberately to cultivate legacies on this plan. Each such hospital will be afforded facilities to use an inch of space and to devote it to a terse intimation of needs and aims. The cost involved will so be trifling, though the publicity should prove ample for the purpose in view. If the plan is generally approved, as it promises to be, we shall hope to develop it in various directions, and so fulfil a useful part by raising the sums accruing to hospitals from legacies during war-time and afterwards. The voluntary hospital system must endure for the protection of the people. It is the privilege of all, who recognise this and know its truth, to lend a hand to strengthen the finances of each institution and in every way to contribute to their financial stability. The points here raised are important, and correspondence is invited.

